# Factors Influencing the Placebo Effect in Patients with Primary Open-Angle Glaucoma or Ocular Hypertension: An Analysis of Two Randomized Clinical Trials

**DOI:** 10.1371/journal.pone.0156706

**Published:** 2016-06-02

**Authors:** Taichi Kawamura, Izumi Sato, Koji Kawakami

**Affiliations:** 1 Department of Pharmacoepidemiology, Graduate School of Medicine and Public Health, Kyoto University, Yoshida Konoe-cho, Sakyo-ku, Kyoto, Japan; 2 Senju Pharmaceutical Co., Ltd., Osaka, Japan; 3 Keihanshin Consortium for Fostering the Next Generation of Global Leaders in Research (K-CONNEX), Kyoto, Japan; National Eye Institute, UNITED STATES

## Abstract

**Objective:**

To explore factors related to the placebo effect in patients with primary open-angle glaucoma (POAG) or ocular hypertension (OH).

**Methods:**

This was a retrospective cohort study of patients with POAG and patients with OH who were treated with placebo. The patients’ data were extracted from two randomized, double-masked, parallel, multicenter clinical trials (trial 1 and trial 2) in Japan. We explored the baseline factors that were associated with the intraocular pressure (IOP)-lowering effect of placebo ophthalmic solution after 4 weeks of instillation treatment at two time points by using multivariable models. The time points were Hour 0 (between 08:30 and 10:30 before instillation) and Hour 2 (within 1.5 to 2.5 h after instillation and by 12:30) at the baseline date and after 4 weeks. The changes in IOP from baseline to 4 weeks at the two time points were evaluated for the IOP-lowering effect induced by placebo instillation.

**Results:**

Of the 330 patients included in the two trials, 89 patients were eligible for the analysis. The results of the multivariable analysis for Hour 0 indicated a high IOP at the baseline date (coefficient: 0.24, 95% confidence interval (CI): 0.02 to 0.46, P = 0.03), and the magnitude of the IOP fluctuation at the baseline date (coefficient: 0.57, 95% CI: 0.24 to 0.90, P = 0.001) was associated with the IOP-lowering effect after 4 weeks. With respect to Hour 2, the trial type was associated with the IOP-lowering effect (coefficient: -1.15, 95% CI: -2.14 to -0.16, P = 0.02).

**Conclusions:**

A large fluctuation in IOP during the day is associated with the IOP-lowering effect induced by placebo in patients with POAG or OH. This finding would be helpful to researchers when designing studies related to glaucoma in the early stages of clinical development of drugs.

## Introduction

Glaucoma is a chronic, progressive optic neuropathy that presents as both optic disc damage and visual field loss [1]. The type of glaucoma is determined by the condition of the iridocorneal angle [2]. Primary open-angle glaucoma (POAG) is associated with an open iridocorneal angle and cupping of the optic-nerve head with corresponding loss of the visual field. Ocular hypertension (OH), which is characterized by an intraocular pressure (IOP) >21 mmHg without clinical signs, can progress into glaucoma if left untreated. The progression of visual field loss in glaucoma is strongly related to IOP, and the reduction of IOP provides solid evidence that the progression of the disease is slowing during treatment [3–6].

Several classes of ophthalmic solutions are available for the medical management of glaucoma (e.g., prostaglandin analogs (PGAs) and beta-blockers) [7]. PGAs are generally used for first line therapy because they have the greatest IOP lowering efficacy.

Various factors cause IOP fluctuations, including diurnal fluctuations, seasonal variations, body position, and stress [8–12]. Moreover, the ranges of these fluctuations in patients with glaucoma are larger than the ranges observed in healthy individuals. A large diurnal fluctuation in IOP is a known risk factor for the progression of glaucoma [13]. For this reason, not only the efficacy of an IOP-lowering agent, but also the degree of diurnal fluctuation is extremely important when considering the appropriate treatment for glaucoma.

In general, a prospective, randomized, placebo-controlled trial is conducted to evaluate glaucoma agents that are in the early stage of development [14, 15]. Although the placebo should be ineffectual in establishing evidence of drug efficacy, placebo effects have been reported in various diseases, including diseases in the ophthalmologic field (e.g., IOP reduction among patients with glaucoma) [16–22]. It is possible that researchers underestimate a drug’s ability to decrease IOP owing to placebo effects, which produce a bias towards the null. Thus, the various factors associated with placebo effects should be considered when selecting study subjects. However, currently, it is unclear which factors are related to placebo effects in patients with glaucoma.

In this study, we aimed to investigate the baseline factors associated with the IOP-lowering effect induced by placebo instillation after 4 weeks of treatment in patients with POAG or OH. In particular, we focused on the patient characteristics, trial type, and IOP fluctuations that were reported in two clinical trials.

## Materials and Methods

### Study design and data source

This study was a retrospective cohort study that utilized data from two randomized, double-masked, placebo-controlled, multicenter, phase 2 clinical trials in Japan (SNJ-2022 [trial 1] [23], SNJ-1656 [trial 2]). Trial 1 and trial 2 were conducted in 2006 and from 2009 to 2010, respectively. These studies were registered in the UMIN-CTR, a clinical trials registry in Japan (UMIN000019142, UMIN000018866).

For trial 1, the key inclusion criteria for the selected subjects were patients with POAG or OH, aged 20 years or older, who had an IOP of ≤31 mmHg and a visual acuity of ≥0.7. After the screening examinations were performed, subjects were required to discontinue any current glaucoma medication for an appropriate length of time as a washout period (e.g., for at least 4 weeks for prostaglandin analogs and beta-blocking agents). After the washout period, subjects underwent additional examinations, and the day the examinations were performed was set as the baseline date. The IOP at the baseline data was measured at two time points (Hour 0 [between 08:30 and 10:30] and Hour 2 [within 1.5 to 2.5 h after the measurement made at Hour 0 and by 12:30]). We refer to Hour 0 and Hour 2 at baseline as “Week 0–0” and “Week 0–2,” respectively. In addition to the above-mentioned inclusion criteria, patients were also required to complete the designated washout at the baseline date, and have an IOP of 18–31 mmHg (for patients with POAG) or 22–31 mmHg (for patients with OH) at Week 0–0. Subjects meeting these inclusion criteria were then randomly allocated to groups that received either 0.1% and 0.15% of SNJ-2022, or placebo twice daily (between 08:30 and 10:30, and between 20:00 and 22:00) in both eyes for 4 weeks. After 2 and 4 weeks, the IOP was measured at Hour 0 and Hour 2. We refer to Hour 0 and Hour 2 after 4 weeks as “Week 4–0” and “Week 4–2,” respectively. The IOP was measured twice at each time point, and the average was used for evaluating the IOP-lowering effect in trial 1. However, in cases in which the difference between the two IOP values was ≥3 mmHg, another IOP measurement was required, and the median value of the three was used for the evaluation. The higher IOP value from among the obtained values for both eyes was selected as the study eye at Week 0–0. If the values were the same, the right eye was selected.

The design of trial 2 was very similar to that of trial 1. The key differences in the inclusion criteria between the two trials were the visual acuity (≥20/40) and the requirement of an IOP confirmation visit prior to the baseline date in trial 2. In trial 2, each subject’s IOP was measured between 08:30 and 10:30 on the confirmation visit after the washout period and on another day within 1 month of the confirmation visit (set as the baseline date if the subject met the inclusion criteria). Subjects who exhibited a change in IOP of ≤3 mmHg were then eligible for the trial. The qualifying subjects were randomly allocated to groups that received 0.003%, 0.01%, or 0.03% of SNJ-1656 or placebo twice daily in both eyes for 4 weeks.

The composition of placebo solution used in study 1 is as follows: sodium chloride, potassium chloride, calcium chloride, magnesium chloride, boric acid, borax, carmellose sodium, sodium chlorite and purified water. On the other hand, the placebo solution used in study 2 was composed of the following: monobasic sodium phosphate dehydrate, sodium chloride, benzalkonium chloride and purified water. The IOP was measured using Goldmann applanation tonometer at all sites in the 2 clinical trials. Both trials were conducted in accordance with the Declaration of Helsinki and were approved by the Institutional Review Board (IRB) of each site.

For the present study, the eligibility criteria were patients who were randomly placed in the placebo group and underwent placebo instillation for 4 weeks in trial 1 or trial 2. The study protocol was approved by the Ethics Committee of Kyoto University Graduate School and the Faculty of Medicine (R0104). All of the patients’ data were certified as anonymous.

### Measurements

Subject characteristics were collected from a Case Report Form that was based on the medical records and questionnaires from the patients in the two indicated trials. These two trials gathered the following information from the patients: age, sex, vital signs, comorbidity, concomitant drug, use of contact lenses, history of ocular surgery, eye examinations (e.g., visual acuity, IOP, visual field), laboratory tests, and adverse events. In the present study, we utilized the following patient information from the trials: age, sex, systolic blood pressure, diastolic blood pressure, pulse rate, comorbidity (hypertension, diabetes, hyperlipidemia, and hyperuricemia), diagnosis (POAG or OH), pretreatment drug for glaucoma, IOP, and time of IOP measurements. The IOP data at Week 0–0, Week 0–2, Week 4–0, and Week 4–2 were collected in this study.

### Outcome

The primary outcome was the factors that were related to the change in IOP from the baseline date to 4 weeks afterward, at the same time point (i.e., a change in IOP from Week 0–0 to Week 4–0 and from Week 0–2 to Week 4–2).

### Statistical analysis

The subject characteristics, including their age, sex, systolic blood pressure, diastolic blood pressure, pulse rate, comorbidities (hypertension, diabetes, hyperlipidemia, and hyperuricemia), diagnosis, pretreatment drug for glaucoma, IOP, and time of IOP measurement, were examined using descriptive statistics. These characteristics were summarized using means and standard deviations for continuous variables and frequencies and percentages for categorical variables. We compared the between-trial subject characteristics (change in IOP from Week 0–0 to Week 4–0 and from Week 0–2 to Week 4–2) using the Mann–Whitney U test.

Simple and multiple regression analyses were performed to explore the baseline factors associated with the IOP-lowering effect induced by placebo instillation. Patient-specific variables (age, sex, and comorbidities), characteristics of the IOP, and trial type (trial 1 or trial 2) were included in the regression model as covariates.

The covariates of the IOP included the baseline IOP (Week 0–0 and Week 0–2) and the difference in the two measurements obtained at Week 0–0 (as noted above, IOP values were continuously measured twice at each time point, and the difference in the two resulting IOP values (Week 0–0 measure 1—Week 0–0 measure 2) was used as a covariate). Moreover, the change in IOP from Week 0–0 to Week 0–2 was included in the model as a covariate of “IOP fluctuation during the day.” The trial type was included to adjust for the differences between the two trials (e.g., design, timing of implementation). Here, we excluded the diagnosis (POAG, OH) from the analysis because it was not related to the change in IOP from Week 0–0 to Week 4–0 or from Week 0–2 to Week 4–2 in the univariate and multivariable regression models. Furthermore, the diagnosis exhibited a moderate correlation with the IOP at Week 0–0 (r = 0.552) and Week 0–2 (r = 0.393).

The results are presented as the partial regression coefficient (PRC) and the corresponding 95% confidence intervals (CIs). P < 0.05 in a two-sided test was considered statistically significant. Data management and statistical analyses were performed using SPSS software, version 22 (IBM).

## Results

**Fig 1:**
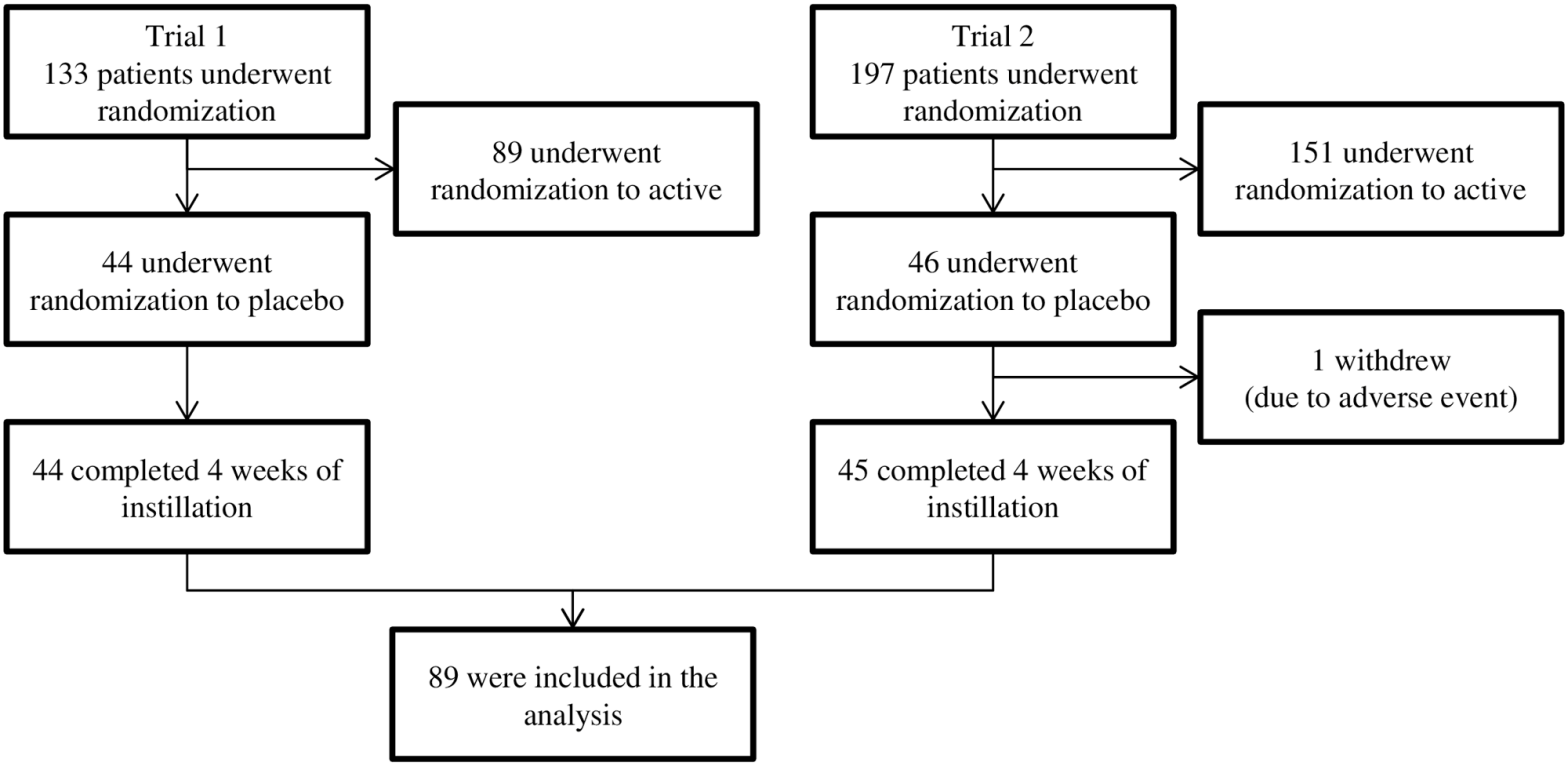
Flow diagram of the subject selection process.

Of the 330 patients included in trial 1 and trial 2, 90 of them (44 in trial 1, 46 in trial 2) underwent randomization to a placebo group. One subject who dropped out of trial 2 due to an adverse event was excluded. Thus, a total of 89 subjects met our study eligibility criteria (Fig 1). Table 1 shows the baseline characteristics of the patients included in the present study. Among the 89 subjects, 58.4% were female, 28.1% had hypertension, 13.5% had diabetes, 21.3% had hyperlipidemia, 51.7% were diagnosed with POAG, and 56.2% had received a pretreatment drug for glaucoma. No large differences were observed between trial 1 and trial 2 regarding the baseline characteristics of the patients.

**Table 1:** Baseline characteristics of study participants (N = 89).

	Trial 1 (N = 44)		Trial 2 (N = 45)		Total (N = 89)	
	N	%	N	%	N	%
**Age, in yrs, mean (±SD)**	**54.6**	**15.9**	**57.5**	**13.0**	**56.1**	**14.5**
**Female sex**	**28**	**63.6**	**24**	**53.3**	**52**	**58.4**
Blood pressure, Pulse rate*						
**SBP, in mmHg, mean (±SD)**	**131**	**18.8**	**128**	**19.1**	**129**	**18.9**
**DBP, in mmHg, mean (±SD)**	**78**	**11.6**	**76**	**11.8**	**77**	**11.7**
**Pulse rate, in pulses/min, mean (±SD)**	**73**	**12.0**	**73**	**12.1**	**73**	**12.0**
**Comorbidity**†						
**Hypertension**	**10**	**22.7**	**15**	**33.3**	**25**	**28.1**
**Diabetes**	**6**	**13.6**	**6**	**13.3**	**12**	**13.5**
**Hyperlipidemia**	**7**	**15.9**	**12**	**26.7**	**19**	**21.3**
**Hyperuricemia**	**0**	**0.0**	**3**	**6.7**	**3**	**3.4**
**Diagnosis**						
**POAG**	**22**	**50.0**	**24**	**53.3**	**46**	**51.7**
**OH**	**22**	**50.0**	**21**	**46.7**	**43**	**48.3**
**Pretreatment drug**	**25**	**56.8**	**25**	**55.6**	**50**	**56.2**
**Prostaglandin analogs**	**21**	**47.7**	**19**	**42.2**	**40**	**44.9**
**Beta-blocking agents**	**6**	**13.6**	**7**	**15.6**	**13**	**14.6**
**Alpha-beta-blocking agents**	**2**	**4.5**	**1**	**2.2**	**3**	**3.4**
**Carbonic anhydrase inhibitors**	**2**	**4.5**	**5**	**11.1**	**7**	**7.9**
**Pilocarpine Hydrochloride**	**0**	**0.0**	**1**	**2.2**	**1**	**1.1**

Abbreviations: SD, standard deviation; SBP, systolic blood pressure; DBP, diastolic blood pressure; POAG, primary open-angle glaucoma; OH, ocular hypertension.

*Value at Week 0–0.

^†^Comorbidity was defined as having both diagnoses and therapeutic medication.

Table 2 describes the IOP and time of IOP measurement at each time point. The mean change in IOP from Week 0–0 to Week 4–0 was 1.8 mmHg, while that from Week 0–2 to Week 4–2 was 1.6 mmHg. The mean change rate from Week 0–0 to Week 4–0 was 7.8%, while that from Week 0–2 to Week 4–2 was 7.2%. Regarding the change in IOP from Week 0–0 to Week 0–2, 18.0% were <0 mmHg, 40.4% were from 0–1 mmHg, 28.1% were between 1–2 mmHg, and 13.5% were ≥2 mmHg. The times of IOP measurement were approximately the same at the baseline date and after 4 weeks. Regarding the differences between the trials, the change in IOP from Week 0–0 to Week 4–0 in trial 2 (1.3 mmHg) was smaller than that in trial 1 (2.2 mmHg) (P = 0.048), and the change in IOP from Week 0–2 to Week 4–2 in trial 2 (1.0 mmHg) was also smaller than that in trial 1 (2.2 mmHg) (P = 0.03).

**Table 2:** IOP values and measurement times at the baseline date and after 4 weeks.

	N	IOP (mmHg)		Change in IOP* (mmHg)		Change rate in IOP† (%)		Time of IOP measurement (time of day)		Time differences‡ (time)	
		Mean	SD	Mean	SD	%	SD	Mean	SD	Mean	SD
**Baseline date**											
**Week 0–0**	**89**	**22.3**	**2.2**					**09:28**	**0:25**		
**Trial 1**	**44**	**22.3**	**2.3**					**09:25**	**0:22**		
**Trial 2**	**45**	**22.2**	**2.0**					**09:31**	**0:28**		
**Week 0–2**	**89**	**21.7**	**2.3**					**11:17**	**0:27**		
**Trial 1**	**44**	**21.7**	**2.5**					**11:16**	**0:22**		
**Trial 2**	**45**	**21.6**	**2.2**					**11:18**	**0:31**		
**Difference between repeated measurements (Week 0–0)**	**89**			**0.2**§	**0.6**						
**Change in IOP from Week 0–0 to Week 0–2(%)**	**89**			**0.6**¶	**1.4**	**2.6**||	**6.2**				
**<0 (mmHg)**	**16**	**(18.0)**									
**0–1 (mmHg)**	**36**	**(40.4)**									
**1–2 (mmHg)**	**25**	**(28.1)**									
**≥2 (mmHg)**	**12**	**(13.5)**									
**After 4 weeks**											
**Week 4–0**	**89**	**20.5**	**2.8**	**1.8**	**2.3**	**7.8**	**10.2**	**09:25**	**0:25**	**0:03**	**0:19**
**Trial 1**	**44**	**20.1**	**2.9**	**2.2**	**2.2**	**9.8**	**9.9**	**09:19**	**0:23**	**0:06**	**0:17**
**Trial 2**	**45**	**20.9**	**2.7**	**1.3**	**2.4**	**5.8**	**10.3**	**09:31**	**0:25**	**0:00**	**0:21**
**Week 4–2**	**89**	**20.1**	**3.0**	**1.6**	**2.4**	**7.2**	**11.2**	**11:19**	**0:26**	**-0:01**	**0:20**
**Trial 1**	**44**	**19.5**	**3.0**	**2.2**	**2.4**	**10.0**	**10.8**	**11:15**	**0:27**	**0:00**	**0:19**
**Trial 2**	**45**	**20.6**	**2.9**	**1.0**	**2.3**	**4.4**	**11.1**	**11:22**	**0:25**	**-0:04**	**0:20**

Abbreviations: IOP, intraocular pressure; SD, standard deviation.

*Change in IOP from Week 0–0 to Week 4–0 (Week 0–0—Week 4–0) and from Week 0–2 to Week 4–2 (Week 0–2—Week 4–2).

^†^Change rate in IOP from Week 0–0 to Week 4–0 ([Week 0–0—Week 4–0]/Week 0–0) and Week 0–2 to Week 4–2 ([Week 0–2—Week 4–2]/Week 0–2).

^‡^Difference in the time of IOP measurement from Week 0–0 to Week 4–0 (time at Week 0–0—time at Week 4–0) and Week 0–2 to Week 4–2 (time at Week 0–2—time at Week 4–2).

^§^Change in IOP between the 1^st^ and 2^nd^ measurements at Week 0–0 (1^st^ measured value—2^nd^ measured value).

^¶^Week 0–0—Week 0–2.

^||^(Week 0–0—Week 0–2)/Week 0–0.

Table 3 shows the associations between the change in IOP from Week 0–0 to Week 4–0 and the subject characteristics. In the univariate analysis, diabetes (coefficient: -1.46, 95% CI: -2.85 to -0.07, P = 0.04) and the change in IOP from Week 0–0 to Week 0–2 (coefficient: 0.65, 95% CI: 0.33 to 0.98, P < 0.001) were significantly related to the change in IOP. In the multivariable analysis, the change in IOP was significantly associated with the IOP at Week 0–0 (coefficient: 0.24, 95% CI: 0.02 to 0.46, P = 0.03) and the change in IOP from Week 0–0 to Week 0–2 (coefficient: 0.57, 95% CI: 0.24 to 0.90, P = 0.001). These results imply that an increase of 1 mmHg at Week 0–0 increased the IOP-lowering effect caused by placebo instillation by 0.24 mmHg, and that an increase of 1 mmHg in the IOP from Week 0–0 to Week 0–2 increased the IOP-lowering effect by 0.57 mmHg.

**Table 3:** Univariate and multivariable regression models at Hour 0.

	Univariate analysis				Multivariable analysis			
	PRC	95% CI		P	PRC	95% CI		P
**Age, in yrs**	**-0.03**	**-0.06**	**0.01**	**0.11**	**-0.00**	**-0.04**	**0.03**	**0.82**
**Female sex**	**0.48**	**-0.51**	**1.46**	**0.34**	**0.75**	**-0.21**	**1.72**	**0.13**
**Comorbidity**								
**Hypertension**	**-0.65**	**-1.72**	**0.43**	**0.24**	**0.19**	**-0.92**	**1.29**	**0.74**
**Diabetes**	**-1.46**	**-2.85**	**-0.07**	**0.04**	**-1.21**	**-2.62**	**0.20**	**0.09**
**Hyperlipidemia**	**-1.14**	**-2.30**	**0.03**	**0.06**	**-0.50**	**-1.74**	**0.74**	**0.42**
**IOP**								
**IOP (Week 0–0)**	**0.22**	**-0.01**	**0.44**	**0.06**	**0.24**	**0.02**	**0.46**	**0.03**
Difference between repeated measurements (Week 0–0)*	**-0.13**	**-0.95**	**0.68**	**0.75**	**-0.55**	**-1.31**	**0.21**	**0.15**
**Change in IOP from Week 0–0 to Week 0–2**†	**0.65**	**0.33**	**0.98**	**<0.001**	**0.57**	**0.24**	**0.90**	**0.001**
**Trial type (Trial 2)** ‡	**-0.85**	**-1.81**	**0.11**	**0.08**	**-0.85**	**-1.75**	**0.05**	**0.07**

R^2^ = 0.296.

Abbreviations: PRC, partial regression coefficient; CI, confidence interval; IOP, intraocular pressure; R^2^, coefficient of determination.

*Change in IOP between the 1^st^ and 2^nd^ measurements at Week 0–0 (1^st^ measured value—2^nd^ measured value).

^†^Week 0–0—Week 0–2.

^‡^The reference group consisted of the subjects in trial 1.

Table 4 presents the associations between the change in IOP from Week 0–2 to Week 4–2 and the subject characteristics. In the univariate analysis, the change in IOP was significantly associated with hyperlipidemia (coefficient: -1.23, 95% CI: -2.44 to -0.01, P = 0.05) and trial type (coefficient: -1.21, 95% CI: -2.19 to -0.22, P = 0.02). In the multivariable analysis, significant associations were noted between the change in IOP and trial type (coefficient: -1.15, 95% CI: -2.14 to -0.16, P = 0.02). Specifically, trial 2 was less likely than trial 1 to reduce the IOP via placebo instillation by 1.15 mmHg.

**Table 4:** Univariate and multivariable regression models at Hour 2.

	Univariate analysis				Multivariable analysis			
	PRC	95% CI		P	PRC	95% CI		P
**Age, in yrs**	**-0.01**	**-0.05**	**0.02**	**0.53**	**-0.01**	**-0.05**	**0.03**	**0.66**
**Female sex**	**0.70**	**-0.33**	**1.72**	**0.18**	**0.69**	**-0.38**	**1.76**	**0.20**
**Comorbidity**								
**Hypertension**	**0.15**	**-0.99**	**1.28**	**0.80**	**0.61**	**-0.61**	**1.83**	**0.33**
**Diabetes**	**-1.07**	**-2.55**	**0.40**	**0.15**	**-0.93**	**-2.48**	**0.63**	**0.24**
**Hyperlipidemia**	**-1.23**	**-2.44**	**-0.01**	**0.05**	**-1.23**	**-2.60**	**0.14**	**0.08**
**IOP**								
**IOP (Week 0–2)**	**0.20**	**-0.01**	**0.42**	**0.07**	**0.23**	**-0.01**	**0.47**	**0.06**
Difference between repeated measurements (Week 0–0)*	**-0.09**	**-0.94**	**0.77**	**0.84**	**-0.49**	**-1.33**	**0.35**	**0.25**
**Change in IOP from Week 0–0 to Week 0–2**†	**-0.34**	**-0.71**	**0.02**	**0.06**	**-0.20**	**-0.61**	**0.20**	**0.32**
**Trial type (Trial 2)** ‡	**-1.21**	**-2.19**	**-0.22**	**0.02**	**-1.15**	**-2.14**	**-0.16**	**0.02**

R^2^ = 0.221.

Abbreviations: PRC, partial regression coefficient; CI, confidence interval; IOP, intraocular pressure; R^2^, coefficient of determination.

*Change in IOP between the 1^st^ and 2^nd^ measurements at Week 0–0 (1^st^ measured value—2^nd^ measured value).

^†^Week 0–0—Week 0–2.

^‡^The reference group was the subjects in trial 1.

## Discussion

This is the first study to use the data from randomized, double-masked, parallel comparison, multicenter clinical trials involving patients with POAG or OH to investigate the factors that influence the IOP-lowering effect induced by placebo instillation. Our results showed that the IOP at Week 0–0 and the change in IOP from Week 0–0 to Week 0–2 were associated with the change in IOP from Week 0–0 to Week 4–0. These associations imply that a higher IOP at Week 0–0 and a large change in IOP from Week 0–0 to Week 0–2 tended to produce a large IOP-lowering effect at Week 4–0 by placebo instillation. Meanwhile, the trial type was associated with the change in IOP from Week 0–2 to Week 4–2.

The diurnal variation in IOP is a well-known phenomenon that occurs both in the eyes of patients with glaucoma and in healthy individuals, with the IOP being high early in the morning and low in the evening [24–26]. Considering that the IOP gradually decreases from morning to noon, the change in IOP that was observed from Week 0–0 to Week 0–2 was likely related to the diurnal variation, thus the patients with large IOP changes between these two measurement times would have large diurnal variations in IOP. Clearly, the IOP in patients with large diurnal variations can easily change at any moment. In addition, the IOP fluctuations that occurred between Week 0 and Week 4 are presumed to be one of the key factors causing the change in IOP after 4 weeks. Indeed, a previous study on glaucoma patients reported that the IOP fluctuated on individual days [27].

Other possible factors for the change in IOP from Week 0–0 to Week 4–0 include the mental stress associated with participating in the clinical trial and the patients’ desire to respond to treatment. First, the lower measured IOP values observed at 4 weeks, relative to those obtained at baseline, may be related to a mental stress-induced increase in IOP at the baseline date. Although the change in IOP from Week 0–0 to Week 4–0 was related to the change in IOP from Week 0–0 to Week 0–2, the change in IOP from Week 0–2 to Week 4–2 was not related to this factor. The reason for this difference may be that the influence of mental stress at Week 0–2 was weaker than that at Week 0–0, as Week 0–2 was the second measurement at the baseline date. The subjects would become habituated to IOP measurement. Thus, the IOP at Week 0–2 might not have included an IOP increase; accordingly, the change in IOP from Week 0–2 to Week 4–2 might be smaller than the IOP change from Week 0–0 to Week 4–0.

Second, the patients’ desire to have a beneficial response to therapy may have also played a role in the IOP-lowering effect at 4 weeks. Indeed, it is well-known that this factor causes a placebo effect [28].

As for why the IOP at Week 0–0 affected the change in IOP from Week 0–0 to Week 4–0, this could be related to regression towards the mean [29]. In fact, the IOP at Week 0–0 was high, while the IOP at Week 4–0 was lower than that at Week 0–0. However, a high IOP at Week 0–2 was not significantly associated with the IOP-lowering effect at Week 4–2, which had a similar tendency as that from Week 0–0 to Week 4–0.

There were several design differences between trial 1 and trial 2; the key difference was the inclusion criteria for the IOP value. In particular, trial 2 applied more stringent criteria than trial 1, in that the change in IOP from the IOP confirmation visit to Week 0–0 had to be within 3 mmHg. Hence, trial 2 excluded those patients with large changes in IOP.

According to the multivariable analysis, the IOP fluctuations that occurred between Week 0 and Week 4 is one of the key factors causing the change in IOP; thus, it is presumed that patients without this factor would experience a lower placebo effect than would patients with this factor. Therefore, as a result of the above noted differences in the trial designs, the number of patients who were at risk of experiencing the placebo effect was likely diminished in trial 2, resulting in less of an IOP-lowering effect. Interestingly, a past clinical trial included a criterion relevant to the difference in IOP between the two measurement dates [14]. Although trial type was not significantly associated with the IOP-lowering effect at Week 4–0, the tendency was similar to that observed at week 4–2. Although, hyperlipidemia affected the change in IOP in the univariate regression models, it is less likely that IOP would be influenced by this factor. This is because hyperlipidemia was not significantly associated with the change in IOP in the multivariable regression model.

Although some constituents of each placebo solution were different, we believe that the IOP would not be influenced by these components as these are normally found in eye drops. The adverse event that caused drop out of the patients in trial 2 was related to placebo instillation. However, the AE was allergic conjunctivitis, which would not influence the evaluation of IOP change in this study.

A previous study on glaucoma patients reported that the regression towards the mean might influence the placebo effect [22]. The results of the present study are not consistent with the findings of a previous study, which showed that setting a separate day entry criterion of IOP did not effectively restrain the placebo effect [20]. However, because the previous study only used averages rather than pulling the data from each patient individually, the obtained results would be expected to differ from those of the current study. In particular, the use of only summaries of published data, rather individual patient data [20], meant the association between the glaucoma patients’ individual characteristics and placebo effects could not be fully ascertained. In this regard, the strength of the present study was the inclusion of individual-level patient data from the clinical trials.

Our study has several limitations. Although the present study was conducted using data from two clinical trials, the sample size was limited. Accordingly, the small sample size limits the generalizability of our results. Secondly, we might not have been able to adjust for all of the potential confounding factors (e.g., personality, lifestyle), as the trials did not necessarily collect information on all of these variables, hence they were not available to us. For example, a previous study reported that changes in blood pressure are positively associated with IOP [30], which might account for the changes in IOP observed in our study. Although we confirmed that the large fluctuations in IOP are related to the low IOP with placebo instillation, it is unclear whether the low IOP could be produced with no treatment, and hence, further research using more variables is needed. A prospective study, especially a crossover study, would be useful to identify the factors related to placebo effect. In the same cohort of patients, a comparison of change in IOP with treatment (viz. active agents, placebo, or no treatment) would clear the relationship between placebo instillation, IOP fluctuation, and IOP change. Nonetheless, the present study has some benefits because this retrospective cohort study with secondary use of data could give results without expenditure of much time and money. Thirdly, our study was conducted using only Japanese patients, which is significant because the IOP is lower in Japanese individuals than it is in Westerners [31]. In several patients, the IOP at the baseline date was less than 21 mmHg, which is the upper limit for normal IOP. Thus, when considering expanding the study to other settings such as the U.S., it might be necessary to analyze patients with higher IOP values. Fourth, this study included patients who had undergone drug treatment for glaucoma and patients who were untreated. Although the patients receiving pretreatment drug for glaucoma had general washout periods in clinical trial before placebo instillation, the IOP-lowering effect induced by placebo might be underestimated in the pretreatment drug recipients. This is because there was a patient who displayed the drug effect for a long period of time even after discontinuation of the drug [32]. Because long washout periods may contribute to disease progression, it might be better to confirm the effect of placebo in patients with untreated glaucoma in future.

## Conclusions

The current study suggests that large fluctuations in IOP are related to the low IOP that is induced by placebo in patients with POAG or OH. This finding may help researchers design studies for glaucoma agents that are in the early stage of development. For example, establishing a visit to confirm diurnal IOP patterns in order to exclude patients with large IOP magnitudes would lead to a more accurate evaluation of the IOP-lowering effect of the drug. As a large difference in effectiveness between an active agent and placebo permits a smaller sample size in clinical trials, this would reduce both the sample size and cost for clinical trials. However, additional studies with large sample sizes and many variables are necessary to expand the generalizability of our study.

## Supporting Information

S1 Data The data set that was analyzed for this study. (XLSX)
